# Computational Drug Repositioning in Cardiorenal Disease: Opportunities, Challenges, and Approaches

**DOI:** 10.1002/pmic.202400109

**Published:** 2025-01-31

**Authors:** Paul Perco, Matthias Ley, Kinga Kęska‐Izworska, Dorota Wojenska, Enrico Bono, Samuel M. Walter, Lucas Fillinger, Klaus Kratochwill

**Affiliations:** ^1^ Delta4 GmbH Vienna Austria; ^2^ Department of Internal Medicine IV Medical University of Innsbruck Innsbruck Austria; ^3^ Comprehensive Center for Pediatrics Department of Pediatrics and Adolescent Medicine Division of Pediatric Nephrology and Gastroenterology Medical University of Vienna Vienna Austria

**Keywords:** bioinformatics, biomedicine, heart disease, renal disease, systems biology, vascular disease

## 1 Background

There is currently increased interest in drug repositioning programs, namely the identification of new therapeutic areas for already approved drugs, both in academia as well as in the biotech and pharmaceutical industry. Since 2012, the number of publications indexed in MEDLINE on drug repositioning or drug repurposing is exponentially increasing with a peak in the year 2021 due to the worldwide search for therapeutic options to combat the COVID‐19 pandemic [1]. Drug repositioning, however, is not new, and pharmaceutical companies have ever since been looking for additional market opportunities for their products, in particular when patents expire and generics manufacturers enter the market of the therapeutic areas of initial approvals [2, 3]. In the pharma world, the term indication expansion is also often used instead of drug repositioning or drug repurposing. In particular for patients suffering from a rare disease who are lacking any approved therapies, drug repositioning represents a very interesting and efficient way of bringing new treatment options to the patient fast [4]. This has also been stressed in a recent position paper from the International Rare Disease Research Consortium [5].

Several international consortia have recognized the trend toward drug repurposing. Two US‐based endeavors focusing on drug repositioning are the Drug Repurposing Hub as well as EveryCure. Researchers from the Broad Institute have created the Drug Repurposing Hub with the aim to construct and curate a library of FDA approved drugs that can be used for systematic drug repositioning screenings [6]. EveryCure's mission is to identify novel treatment options for patients with rare diseases via computational drug repositioning. Two European initiatives in the context of drug repositioning are the Repo4EU (https://repo4.eu/) and the REMEDi4ALL (https://remedi4all.org/) consortia, both being public–private partnerships with the aim to develop tools for computational drug repositioning but to also apply these tools and develop novel therapeutic options for selected indications. Next to the worldwide drug repositioning effort in the context of COVID‐19, there are at least three additional reasons why drug repositioning programs are gaining momentum. First, the molecular characterization of disease processes is continuously improving, and we understand more about key molecular pathways and disease‐modifying proteins, forming the basis to find drugs counterbalancing these dysregulations on the molecular level. Second, the arsenal of computational tools, methods, and workflows is getting better at matching disease pathobiology and drug mechanism of action (MoA), identifying novel connections and thus potential targets for therapeutic intervention. And third, the list of successful repositioning cases is getting longer. Even the current blockbuster drugs of GLP1 agonists can be seen as positive drug repositioning examples, both scientifically and commercially. Initially being developed for the treatment of diabetes mellitus, drugs from this compound class are in the meantime also being approved for the treatment of obesity and are in clinical development for several indications across different therapeutic areas.

In this viewpoint article, we will discuss (i) computational and experimental approaches to discover repositioning opportunities, (ii) challenges in the further development of the discovered compounds, and (iii) repositioning approaches in the context of kidney and cardiovascular disease (CVD).

## 2 Approaches to Discover New Repositioning Opportunities

**FIGURE 1:**
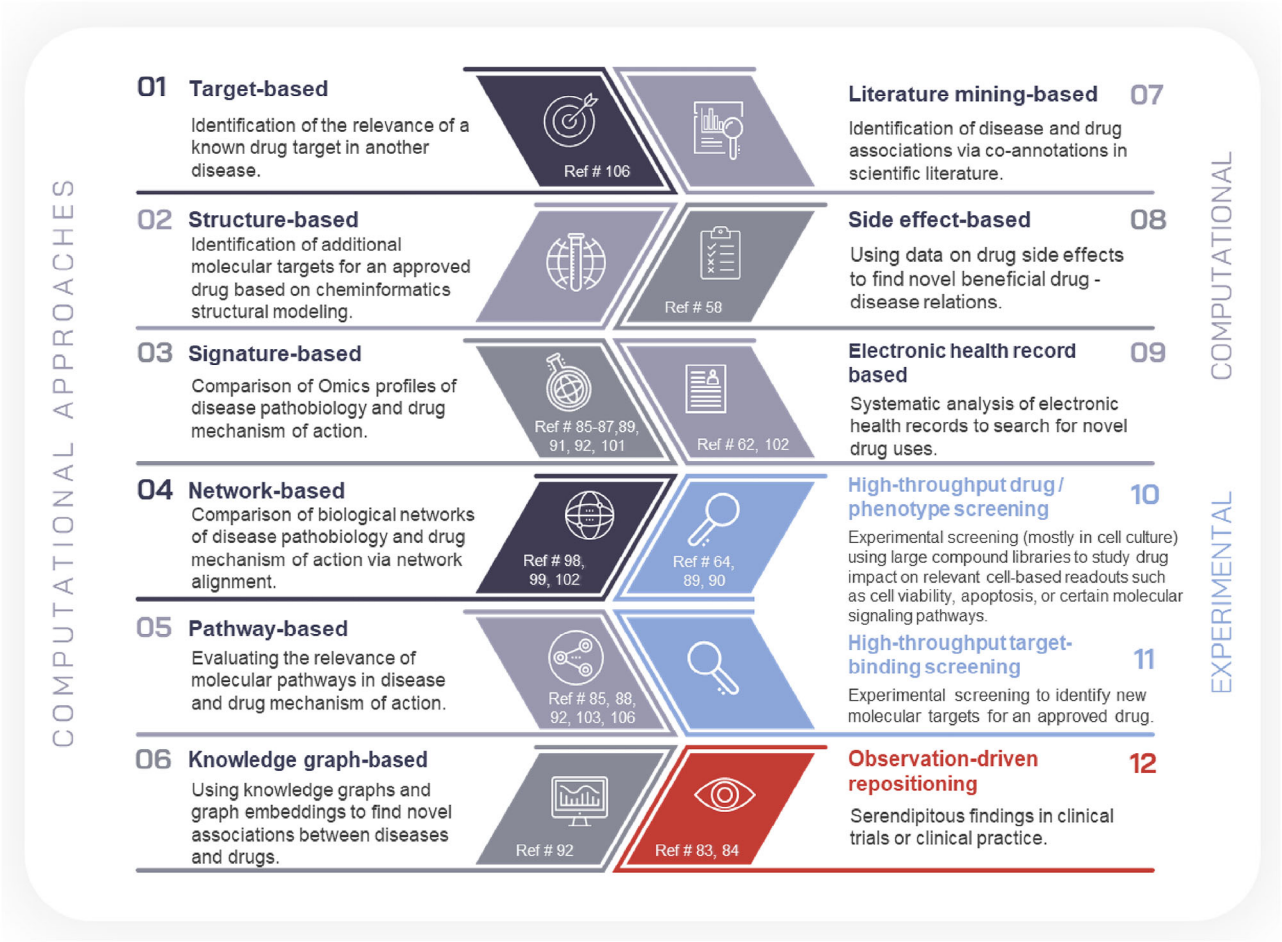
Overview on drug repositioning approaches.

Next to observation‐driven drug repositioning in the context of clinical trials or clinical practice as well as experimental methods such as binding assays or experimental phenotype screens, several computational methods and approaches have been developed to systematically search for new drug repositioning opportunities. These computational approaches make use of information on direct drug targets, affected molecular pathways and biological mechanisms, drug side effects, omics signatures on disease pathobiology and drug mechanism of action, but also on data from clinical trials or electronic health records (EHRs) from patient registries [7, 8, 9, 10]. Key computational and experimental approaches as given in Figure 1 will be discussed in the following sections.

### 2.1 Computational Drug Repositioning Approaches

#### 2.1.1 Target‐Based Repositioning

A major challenge and one of the most crucial early steps in drug discovery and development is the selection of an appropriate drug target. For the majority of approved drugs, the direct drug target is known, however, not always being responsible for the drug's full efficacy potential. Information on a drug's direct drug target can nevertheless be used in target‐based drug repurposing by identifying additional indications in which the drug target is causally linked with disease development and/or disease progression [11]. Databases holding information on direct drug‐target relations include, for example, DrugBank [12], the Therapeutic Target Database [13], or STITCH from the EMBL‐EBI [14].

Two open‐source tools aiming to consolidate information from different sources to associate drug targets to diseases are the Open Targets platform [15] and Pharos [16]. Open Targets is a comprehensive platform that integrates data types such as genetic associations, gene expression data, and pathway information, as well as results from literature‐mining approaches to systematically identify and prioritize therapeutic drug targets in the context of human diseases. The development of Open Targets is driven by researchers from EMBL‐EBI and is a collaboration between scientific institutions and pharmaceutical companies. Users have the option of either evaluating individual proteins (potential drug targets) or searching for potential novel drug targets for a selected disease (phenotype) [15]. Pharos is a web application consolidating information on drug targets in the context of human diseases and has been established by researchers from the “Illuminating the Druggable Genome” consortium [16]. Like in Open Targets, users can either search for a target (protein) or a disease and are presented with lists of ranked diseases or potential target proteins, respectively. Protein targets are furthermore classified into four categories, namely Tclin (targets, that have at least one approved drug), Tchem (targets, that have a compound with an activity cutoff <30 nM), Tbio (targets, that have either a high annotation score based on scientific literature or antibodies targeting this protein), and Tdark (targets without known drugs or compounds targeting these proteins and with low annotation scores based on scientific literature). Especially the proteins in the Tclin category are prime candidates for target‐based drug repositioning approaches.

Si et al. recently identified a set of promising novel drug targets in the context of chronic kidney disease based on the analysis of proteomics and transcriptomics data in combination with Mendelian randomization analysis and investigation of protein–protein interactions as well as colocalization analysis of protein‐coding genes [17]. These drug targets might pave the way for the discovery of drugs with the potential to beneficially impact the course of disease for patients with chronic kidney disease. Fu et al. used a similar approach based on Mendelian randomization to evaluate the relevance of drug targets from diabetes medications in the context of osteoarthritis [18]. They propose thiazolidinediones as promising disease‐modifying agents in osteoarthritis due to the predicted role of the direct drug target PPARG in the context of osteoarthritis progression.

#### 2.1.2 Structure‐Based Repositioning

Information on the protein drug target is also key in structure‐based repositioning approaches. Structure‐based methods are more cheminformatics than bioinformatics‐driven and leverage structural similarities of drugs to identify compounds with the potential to target the same proteins as already approved drugs. Known pharmacokinetic profiles and safety data can also be used in this approach. Structure‐based methods are broadly classified into traditional and advanced AI/ML‐aided approaches which use high‐quality structures of receptor proteins and ligands. Traditional approaches are based on high‐throughput screenings of compounds in vitro, whereas the application of machine learning algorithms in the computational process facilitates docking simulations, increasing the accuracy and efficiency [19].

#### 2.1.3 Omics Signature‐Based Repositioning

The core strategy of this approach in contrast to the target‐based and structure‐based repositioning approaches is not only to focus on a single protein but also to compare whole omics signatures (i.e., gene expression patterns or protein abundance changes) in a given disease with the omics signatures of various drugs to identify those with the potential to reverse the disease signature [20]. A landmark study on this approach was published in 2006 by Lamb et al. making use of gene expression profiles of 164 compounds to identify relations between drug mechanism of action and dysregulations in disease on the transcriptome level, a dataset they called Connectivity Map [21]. In the meantime, the initial set has been extended to thousands of gene expression profiles for thousands of different compounds that can be used for transcriptomics signature‐based drug repositioning, a process which is currently also known as “connectivity mapping”. To generate in total over 1 million transcriptomics profiles, researchers focused on 1000 landmark genes that encode for over 80% of the variation in biological data on the transcriptional level, thus creating the L1000 dataset [22]. This dataset is integrated with other resources within the Library of Integrated Network‐based Cellular Signatures (LINCS) program, including proteomic, metabolomic, and epigenomic data [23]. The data is publicly available and accessible via the iLINCS web‐based platform [24].

There are currently several other tools and web applications available for signature‐based drug repositioning. Mergeomics 2.0 is a web‐based platform and open‐source R package for integrating multi‐omics data to reveal insights into biological pathways, networks, and key drivers important to disease pathogenesis and ultimately predict therapeutic applications [25]. A new functional module called PharmOmics matches multi‐omics‐informed disease pathways or networks with drug signatures to predict potential therapeutic drugs [26]. The platform is designed to enable the analysis of summary statistics of multiomics data, gene sets, and biological networks. PharmGWAS is a database that leverages genome‐wide association studies (GWAS) to identify (i) candidate drugs for repurposing, (ii) drug combination therapies, (iii) drug resistance, or (iv) side effects across a broad range of diseases. GWAS datasets have been retrieved from biobanks and consortiums, and PharmGWAS deposited compound‐perturbed signatures retrieved from CMap2.0 and gene expression signatures from SigCom LINCS [27]. A Single‐cell Guided Pipeline to Aid Repurposing of Drugs (ASGARD) uses scRNA‐Seq data from disease samples and attempts to identify drugs with the highest potential in the treatment of the respective disease [28].

#### 2.1.4 Network‐Based Repositioning

This approach aims to model complex biological systems on the level of biological networks to analyze interactions among different entities, such as proteins, diseases, or drugs. By mapping functional interactions onto a network, these methods enable measurements on the connectivity, proximity, or cluster formation of these entities, for example, within protein–protein interactions, gene regulatory networks, and biological pathways, to allow predictions and to enable in silico drug repurposing.

Guala and Sonnhammer tested network crosstalk‐based drug repurposing approaches and constructed a benchmark for performance assessment of network‐based drug repurposing tools [29]. To evaluate the cross‐talk between sets of drug targets and disease‐related genes, they calculated four distinct measures. They evaluated the shortest path between every drug target and its closest disease gene [30], as well as three separate scores to estimate crosstalk based on the number of links between two sets of nodes [31, 32, 33]. Sadegh et al. proposed an open‐source platform (NeDRex) for network‐based drug repurposing that integrates gene, protein, drug, drug target, and disease annotations as well as their relationships [34]. The NeDRex platform incorporates a number of state‐of‐the‐art network algorithms and a case study exemplified the broad applicability of the platform by extracting meaningful ovarian cancer disease pathways from starting seed nodes. The obtained disease model contained newly identified connector genes which, together with the seed genes, participate in relevant pathways that could not be retrieved using the seed genes alone. Maier et al. developed the Drugst.one platform that provides user‐friendly, web‐based utilities with interactive network visualization capabilities [35]. By integrating 14 data sources covering protein/gene, drug, and disease entities, the tool can enrich a given list of proteins/genes with clinically relevant information such as targeting drugs or disease associations. Other use cases include the integration of known pathways to display adjacent diseases and drugs, or to project gene expression data on the proteins in the network. Yang et al. proposed with DRONet a framework to make use of effectiveness comparative relationships by combining network embeddings, generated from a heterogeneous drug‐disease network, and ranked learning to predict drug effectiveness by ranking drugs based on their therapeutic efficacy for specific diseases, utilizing methods like RankNet, LambdaRank, and LambdaMART [36]. Advances in DNA/RNA sequencing have enabled the development of algorithms for drug repurposing by targeting disease models derived from sequencing profiles mapped to the human protein–protein interactome network. Cheng et al. proposed the Genome‐wide Positioning Systems network for drug repurposing based on whole‐exome sequencing and transcriptome profiles from approximately 5000 patients across 15 cancer types taken from The Cancer Genome Atlas, prioritizing new indications for approved drugs [37]. Validation showed that the approved drug ouabain, used for cardiac arrhythmia and heart failure, exhibited significant antitumor activity in lung adenocarcinoma pathways. Moreover, another study by the same group used network proximity within the human interactome to identify hundreds of new drug–disease associations [38]. They validated their predictions against large‐scale patient data, supported by mechanistic in vitro data, demonstrating an increased risk of coronary artery disease with carbamazepine and a decreased risk with hydroxychloroquine, compared to levetiracetam. This highlights the potential of human interactome‐based methods in uncovering new therapeutic uses for existing drugs and understanding their broader impact on disease pathways. Fiscon et al. stated that molecular footprints of diseases are not randomly scattered across genes, but colocalized in highly interconnected subnetworks [39]. For a drug to be effective against a specific disease or to predict off‐target adverse effects, drug targets should be proximal to a disease module. The study compared different frameworks using five different proximity metrics, evaluating complex human diseases such as breast and prostate neoplasms, schizophrenia, and liver cirrhosis. Ruiz et al. introduced the multiscale interactome, a method that models a network of disease‐perturbed proteins, drug targets, and biological functions [40]. Their proposed network is comprised of 17,660 human proteins, 9798 biological functions, 1661 drugs, and 840 diseases. By performing random walks in the network, the method evaluates the propagation of indirect drug effects to biological functions and protein–protein interactions, enabling the prediction of drug–disease treatments, the identification of proteins and biological functions related to treatment and the prediction of genes that alter a treatments efficacy and adverse reactions. The method was validated by comparing drug diffusion profiles to gene expression signatures from the Connectivity Map [21].

Network‐based repositioning approaches have been used by different research groups in various therapeutic areas such as atopic dermatitis [41], COVID‐19 [42], non‐alcoholic fatty liver disease [43], metabolic syndrome [44], but also in cardiorenal disease as discussed later in the specific section on drug repositioning examples in cardiorenal disease.

#### 2.1.5 Pathway‐Based Repositioning

The core strategy of this approach is to focus on the impact of drugs on molecular pathways rather than just the direct drug target leveraging information on disease and drug‐specific pathway dysregulations. Drug REpurposing based on BIOlogical Pathways (DREBIOP) is one of the exemplary concepts that comprehensively utilizes information from different sources to evaluate drug repurposing cases where the drug's effect is mediated through biological pathways [45]. The authors exemplify the beneficial impact of ergocalciferol for rickets based on its interference with the vitamin D pathway. Another example of pathway‐based drug repurposing is the identification of new therapeutic opportunities for breast cancer subtypes through targeting driver pathways to overcome resistance to treatment [46].

#### 2.1.6 Knowledge Graph (KG)‐Based Repositioning

Knowledge‐based approaches enhance network‐based methods by incorporating domain‐specific knowledge into graph‐based structures, enabling machine learning tasks and providing deeper insights into biological interactions. Graph embeddings further advance this by mapping multi‐modal nodes, relationships, or entire sub‐graphs into low‐dimensional vectors.

Himmelstein et al. modeled a complex graph incorporating 11 biological entities and 24 relationship types, using data from 29 public resources that cover compounds, diseases, genes, anatomies, pathways, pharmacologic classes, side effects, and symptoms, among other entity types [47]. The method could identify paths correlating with compound–disease pairs previously shown to be effective. Additionally, a machine learning approach predicted novel treatments as compound–disease relationships, which were validated against new indications from DrugCentral and clinical trial data. A case study identified eight promising drug repurposing candidates for epilepsy. Jain et al. address the issue of insufficient disease specificity in their work [48]. Diluted information and coarse clustering of disease nodes limit the ability to identify new targets or drug synergies. Therefore, they propose a method to create hypergraphs, in which hyperedges encode biological pathways. For this, pathways are converted into embeddings using a modification of the node2vec algorithm. The predictions were compared to the multiscale interactome, a state‐of‐the‐art KG. Seven promising repurposing candidates were identified for Alzheimer's disease. Ghorbanali et al. address the challenge of integrating homogeneous drug and disease features along with negative data into a unified latent space [49]. The method utilizes embeddings to predict unknown associations in a sub‐graph. Prediction area under the curve scores of ∼90% was achieved. Additionally, potential drugs for coronavirus infection and skin‐related diseases were predicted, with several drug candidates for skin‐related conditions having previously demonstrated efficacy in other studies. Santos et al. provide an infrastructure to facilitate automatic analysis, visualization, and knowledge extraction [50]. The framework incorporates 26 biomedical databases, including experimental omics data, public databases, and literature. It is built as an open‐source graph‐based platform to enable precision medicine and clinical decision‐making. Machine learning and graph algorithms are used to enhance information or to predict links in the graph. Its capabilities were validated by demonstrating a significantly higher expression of CT45 in serous ovarian adenocarcinoma, confirming its role as a biomarker for long‐term survival. Amiri Souri et al. proposed with DT2Vec+ a method to identify novel drug‐target interactions via link predictions and network embeddings [51]. The model shows promising results in predicting the degree and type of interactions and proposing potential novel drugs to target cancer‐specific biomarkers. The work of Lobentanzer et al. aims to unify the fragmented landscape of biomedical knowledge and to simplify the process of creating task‐specific KGs [52]. Biocypher offers a framework that focuses on modularity, reproducibility, harmonization, reusability, and accessibility. It builds on top of rigid standards and allows exchanges, modifications, and extensions by utilizing ontologies to map data to concepts. Multiple case studies act as proof of concept to demonstrate practical use, for example, by enabling federated learning in the Care‐for‐Rare project, where multiple children's hospitals collaborate worldwide to train a shared model while keeping the data decentralized and private by only using locally employed, task‐specific KGs and by sharing only the model's configuration parameters and anonymous results.

KGs are an active research area and hold the potential when combined with ML methods to unravel novel indirect associations between diseases and drugs without any direct associations between the disease and the drug already reported in scientific literature.

#### 2.1.7 Literature Mining‐Based Repositioning

As the number of scientific articles in MEDLINE has almost tripled in the last decade and continues to grow by over 850,000 abstracts annually, extracting biomedical information from scientific literature increasingly relies on automated text mining and natural language processing methods. Deep learning technologies such as long short‐term memory, convolutional neural networks, or bidirectional encoder representations from transformers have shown the potential of forming associations between genes, proteins, and drugs [53]. Challenges include abbreviations and ambiguity of terms, for example, gene symbols that are identical to common phrases or unspecific sub‐strings that are falsely recognized as part of longer, more specific entries. In contrast, rule‐based methods allow the definition of more stringent patterns generated by domain experts. Although this approach involves manual curation and longer development cycles, it can significantly enhance overall precision for certain entity types, such as proteins [54]. The overall aim of these literature mining approaches is to extract high‐quality associations and ideally causal relations between genes/proteins, diseases, and drugs that can be used to form new links between drugs and diseases via intermediate genes/proteins. A commonly used resource storing associations between genes/proteins and diseases from literature mining approaches is DisGeNet [55].

#### 2.1.8 Side Effect‐Based Repositioning

Similarities between drugs are identified based on side effect profiles in this approach. Kumar et al. used side effect profiles to search for additional drugs with a potential beneficial impact on epilepsy development and progression. They identified paroxetine as a compound with a potential beneficial impact on seizures [56]. Data on side effects have been extracted from the SIDER database [57], which however seems to be no longer maintained as the last data update dates back to 2015. Another resource holding data on adverse events is the FDA adverse event reporting system that is in contrast to SIDER still regularly updated. Paci et al. developed a method to measure similarities and to reposition drugs for CVDs also making use of side effect data among other datasets [58, 59]. They formulated disease and side‐effect networks to evaluate the proximity of potential drug candidates to diseases that are distal to side effects.

#### 2.1.9 Electronic Health Record (EHR)‐Based Repositioning

This approach leverages the vast amounts of real‐world clinical data captured in EHRs, including patient demographics, diagnoses, treatments, outcomes, and lab results, to explore correlations between drug treatment and positive outcomes that are related to conditions other than the one for which the drug was originally approved [60]. Retrospective analyses of EHR data can on the one hand lead to the de‐novo identification of drug repositioning opportunities but can on the other hand also be used to validate findings from other computational prediction methods. Metformin, a drug primarily used as therapy in diabetes, for example, was found to reduce the risk of dementia in patients with type 2 diabetes after statistical analysis of two large‐scale EHR databases [61]. Deep‐learning methods have been developed to emulate clinical trials and predict the effect of drugs based on retrospective analysis of large EHR datasets as shown by Liu et al. in the context of coronary artery disease [62]. The UK Biobank is a valuable research resource holding biomedical information on around half a million UK participants in deidentified form [https://www.ukbiobank.ac.uk/]. Data are accessible for approved research groups and publications reporting findings based on analyses of UK Biobank data are exponentially increasing over the last 10 years. The UK Biobank represents an attractive resource for addressing research questions beyond drug repositioning, including the exploration of adverse events, the identification of novel drug targets, and the investigation of comorbidities in selected patient groups.

### 2.2 Experimental Repositioning Approaches

Main experimental drug repositioning approaches include (i) high‐throughput binding assays to identify new targets for approved drugs and (ii) high‐throughput phenotype screenings. These methods screen existing compounds against a wide range of biological targets or disease models in a systematic and automated manner. High‐throughput target‐binding screening approaches rely on the effects of existing drugs on cellular or animal models of different diseases without prior knowledge of specific targets. The aim is to identify drugs that interact with these targets and potentially modulate disease pathways [63]. Phenotype screenings are usually performed in cell‐based assays determining the impact of a number of compounds on cell‐based readouts such as cell viability, apoptosis, cell motility, cell morphology, or using assays to monitor certain signaling pathways. Asawa et al., for example, tested the impact of around 8000 compounds on cell viability of polycystic kidney cells in vitro to identify compounds with an anti‐proliferative effect in a search for potential novel treatment options for patients with polycystic kidney disease [64].

### 2.3 Observation‐Driven Repositioning (Serendipitous Findings)

Observations in clinical trials that can be considered as serendipitous findings play a significant role in the field of drug repositioning and have led to the development of blockbuster drugs in the past [65]. These include (i) the prominent example of sildenafil which was initially developed for systemic hypertension but was later on developed for the treatment of erectile dysfunction due to observed beneficial “side effects” in clinical trials [66], (ii) minoxidil which has initially been developed to treat hypertension with its further application for inducing hair regrowth in alopecia areata [67], or also (iii) amantadine which has initially been approved for influenza and is now also clinically used to treat symptoms of Parkinson's disease [68].

Most successful repositioning cases until now were observation‐driven or based on serendipitous findings. Accessible datasets like the UK Biobank, that also include omics datasets, have paved the way for systematic data‐driven drug repositioning approaches. It is still too early to determine which of the computational biology‐driven approaches has the highest probability of success in identifying viable drug repositioning opportunities and eventually it will be a mix of complementary methods that should be applied to increase chances of success. Figure 1 holds references to conducted drug repositioning approaches in the context of cardiovascular and renal disease.

No matter by which method new drug repositioning opportunities are discovered, the initial discovery is just the first step on the way of bringing a new therapy into the clinic and to the market as will be discussed in the next chapter.

## 3 From Discovery to Market

**FIGURE 2:**
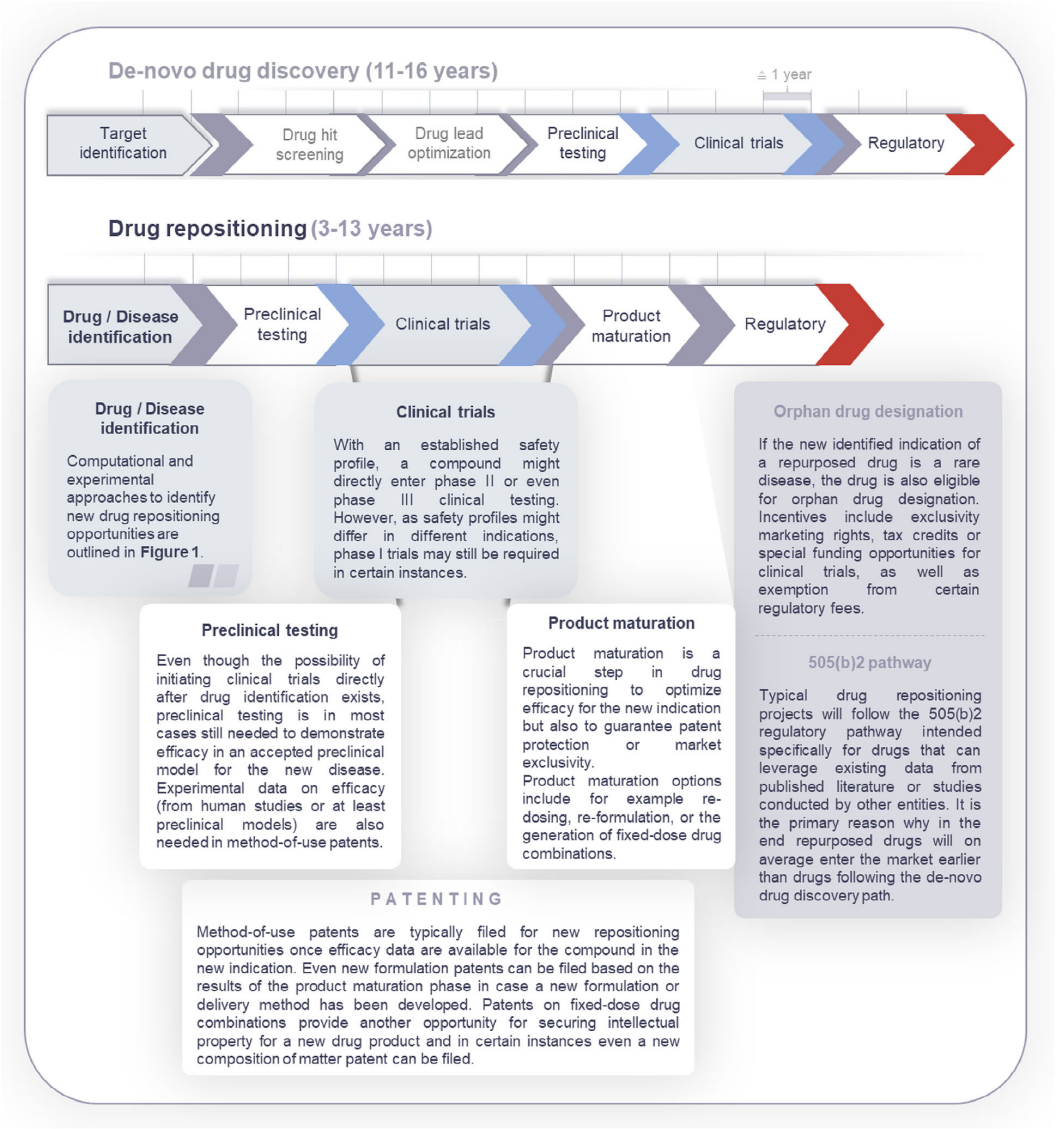
Challenges on the way to a new approved repurposed drug.

Repositioning is often described as a shortcut to reduce the time to market for a drug and drastically decrease the development costs. Widely used figures in the drug discovery area report that it takes 10000 candidates to start with to end up with 1 drug on the market and that this process takes on average 10–17 years and comes with a mean price tag between 1.6 and 2.8 billion USD [69, 70]. The exact savings achieved by drug repurposing in time, risk, and money can be unclear, with some conflicting evidence. Some reviews suggest that about 30% of repurposing efforts are successful and lead to a product approved for marketing, compared to about 10% for new drug applications (NDAs) in general, while other studies argue that repurposed drugs do not necessarily have higher success rates than new drugs, with efficacy often being the limiting factor rather than safety [71]. Once a candidate compound for repurposing has been discovered, the path to clinical utilization and marketing remains a cost‐intensive challenge, often referred to as the “valley of death” between basic and clinical research [72]. Numerous experts state that the benefit of repurposing lies in the availability of an established safety profile and that for these compounds preclinical animal models to test safety and even clinical (safety) trials may be skipped up to Phase II or even Phase III. There may be criteria, based on which such a shortcut seems straightforward and even obvious, such as: the drug was shown to be safe in multiple human studies, whereas the animal model does not adequately recapitulate the human disease for which there is substantial unmet medical need, data from in vitro or in silico experiments are supportive, and dosing and administration are consistent with prior human experience for the therapeutic agent of interest [73]. However, in many cases either funders or regulatory authorities may still require the generation of partly redundant data, thereby reducing potential cost savings. Major steps and key concepts in the drug repositioning development path are shown in Figure 2.

A significant factor along this way is patentability of the repurposed drug candidates which is crucial to ensure market exclusivity and protect intellectual property. Only when some form of patent protection or market exclusivity can be guaranteed, the costs of providing the necessary preclinical and clinical data to obtain marketing authorization can be earned back. De Visser et al. discuss the challenge of pricing in drug repositioning, with some compounds having the opportunity of gaining a monopoly leading to exorbitantly high prices as seen for colchicine in the US whereas other compounds are hardly reimbursed to an extent that makes it attractive for companies to invest in drug repurposing in the first place [74]. The authors further advocate government policies to adapt the regulations of appropriate and/or exclusive reimbursement to make drug repurposing more attractive and more predictable for companies. Although better definitions and appropriate pricing models may be introduced by regulators in the future to avoid such “hijacking” while providing a predictable case for companies to (co)‐invest in drug repurposing, currently protection is the most promising way to secure a business case for repurposing. Without any incentives, compounds, which could be potentially repurposed, usually do not make it past the academic exercise of identifying their potential alternative use. Although novel molecules, so‐called new chemical entities, are typically patented on the substance level, repurposed drugs more often rely on the protection of the product (formulation, composition, route of administration, etc.), the method of use (indication), or a combination of two or three of the before mentioned categories. Second medical use patents, sometimes also referred to as Swiss‐type patents, typically involve claims like “substance X for the treatment of condition Y”. Novelty and non‐obviousness are the two main criteria by which a repurposed drug is deemed patentable [75], leading to the situation that many potential therapies which would rely on a repurposed compound are mentioned—sometimes even in a speculative fashion—in research articles and are thus either obvious to the expert or at least not novel anymore. According to the current situation regarding necessary IP protection to cover the costs of developing these drugs, these compounds are basically burned ground and will (excluding self‐medication or supported by non‐profit programs) likely never make it to the patient.

Additional concepts may help especially in the case of repurposing. Formulation patents can be obtained for new formulations of existing drugs, such as extended‐release versions or new delivery mechanisms. Combination patents can be filed for new combinations of existing drugs that provide a synergistic effect. If the repurposed drug involves a new chemical entity or a novel combination of active pharmaceutical ingredients, even a new composition of matter patent can be issued. In some regions, supplementary protection certificates (SPCs) can extend the patent life of a drug beyond the usual term, providing additional market exclusivity [76], which is especially attractive for the current owner or manufacturer of a drug to perform indication expansion as part of the business strategy to explore new markets, or as part of the life‐cycle management of a drug product. Libraries of compounds that are available for repurposing can be divided into immediately available “on‐market” drugs which are either still under active patent or exclusivity protection (“on‐patent” drugs) or where this protection has expired (“off‐patent”). Off‐patent drugs are the prime group for repurposing efforts, after which they at some point become “off‐market” drugs that have been discontinued, in some cases due to safety concerns, rendering them less ideal for repurposing [77]. For drugs repurposed to treat rare diseases, obtaining orphan drug status can provide market exclusivity for a certain period, along with other incentives.

The Orphan Drug Designation Pathway is designed to encourage the development of drugs for rare diseases, which affect a small percentage of the population [78]. To be eligible, the drug must be intended to treat a rare disease or condition affecting fewer than 200,000 people in the U.S., or it must be unlikely to recover the costs of development and marketing [79]. Additionally, the drug must provide a significant benefit over existing treatments. Incentives for this pathway include 7 years of exclusive marketing rights in the U.S. after approval, tax credits up to 25% of the clinical trial costs, funding for clinical trials, and exemption from certain FDA fees.

The 505(b)(2) pathway is a streamlined process for NDAs that allows for the use of existing data [https://www.fda.gov/media/156350/download]. It is also eligible to rely on data not developed by the applicant for the application, such as published literature or studies conducted by other entities. This pathway is used for drugs that are modifications of existing drugs, such as new formulations, combinations, or new indications. Incentives include reduced development time by leveraging existing data, which can significantly shorten the development timeline, and cost savings due to the reduced need for extensive clinical trials.

Although the Orphan Drug Designation is specifically for rare diseases and is not limited to repurposed compounds, the 505(b)(2) pathway is for any drug that can leverage existing data, thus focusing on repurposed drugs. Orphan drugs receive specific incentives like market exclusivity and tax credits, which are not inherent to the 505(b)(2) pathway. Both pathways involve rigorous FDA review processes to ensure safety and efficacy, and both can utilize existing data to support the application, though the 505(b)(2) pathway explicitly allows for this reliance. These pathways provide valuable mechanisms to bring repurposed drugs to market more efficiently, addressing both rare and common conditions.

As part of the IP protection efforts, or due to specific requirements of the alternative indication, product maturation involves optimizing the repurposed drug's formulation, dosing, and delivery to enhance its efficacy, safety, and marketability [80]. Re‐dosing involves optimizing the dosage to achieve the best therapeutic effect with minimal side effects, which may require new clinical trials to determine the optimal dose for the new indication. It also includes developing new dosing schedules, such as once‐daily or extended‐release formulations, to improve patient compliance and outcomes. Re‐formulation focuses on creating new formulations, such as oral, injectable, or transdermal, to improve drug delivery and patient convenience. It also involves reformulating the drug to enhance its stability, shelf‐life, and bioavailability. Drug combinations aim to achieve synergistic effects by combining the repurposed drug with other drugs, enhancing therapeutic outcomes [81]. This also includes developing fixed‐dose combination products that simplify treatment regimens and improve adherence [82]. Companion diagnostics are developed to identify patients who are most likely to benefit from the repurposed drug, enabling personalized medicine. This involves identifying biomarkers that can predict response to the drug, allowing for more targeted and effective treatments. Challenges include the requirement for regulatory approval of each new formulation, dosing regimen, or combination, which may involve new clinical trials and data submissions. Finally, ensuring market acceptance involves gaining the approval and adoption of new formulations and combinations by healthcare providers and patients [4].

## 4 Selected Examples From the Cardiorenal Space

For almost two decades, antihypertensive medication has been the only treatment regimen with renoprotective effects. This has significantly changed in the last few years with new drugs from different drug classes showing good results regarding renal but also cardiovascular outcomes. These mainly include sodium glucose cotransporter‐2 inhibitors (SGLT2i), non‐steroidal mineralocorticoid receptor antagonists (MRAs), selective endothelin receptor antagonists (ERAs), and also glucagon‐like‐peptide 1 receptor agonists (GLP1RAs) [83, 84]. All these drugs can be considered as examples for drug repositioning or indication expansion as they have initially been developed for other diseases, in the case of SGLT2i and GLP1RAs diabetes mellitus. Current research efforts are focusing on identifying responders for these new drugs to optimize treatment and update therapy guidelines in the context of cardiorenal disease. A better understanding of disease pathophysiology and the identification of predictive clinical and molecular markers are essential for this task. A better understanding of the molecular mechanisms of disease is also crucial for identifying novel therapeutic targets as well as additional novel treatment options.

The finding that the JAK/STAT signaling pathway is activated in diabetic kidney disease (DKD) progression based on the analysis of omics data for example has led to the investigation of the JAK inhibitor baricitinib. Baricitinib beneficially impacted albuminuria levels in patients with type 2 diabetes and DKD in a Phase II clinical trial [85]. Baricitinib in addition reduced levels of inflammatory markers such as CCL2, TNFR1/2, ICAM1, or serum amyloid A, however, leading to a higher number of patients experiencing episodes of anemia. It is unclear whether this was the primary reason why no follow‐up studies have been conducted for baricitinib in DKD. Anti‐inflammatory drugs have also been identified as potential novel therapeutic options for DKD in a transcriptomics signature‐based drug repositioning approach by Klein et al. making use of drug expression profiles being available in the Connectivity Map [86]. Dimethylaminoparthenolide, a water‐soluble analogue of parthenolide, has been selected for preclinical validation in vivo and had a beneficial impact on the degree of glomerulosclerosis and tubulointerstitial fibrosis. Connectivity mapping has also been used to identify compounds of interest to tackle glomerulopathies. Chung et al. consolidated publicly available transcriptomics signatures including data from focal segmental glomerulosclerosis (FSGS) patients, minimal change disease patients, and IgA nephropathy patients and identified a set of drugs beneficially interfering with endoplasmic reticulum stress and unfolded protein response, mechanisms that they have identified as relevant in disease progression. The EGFR inhibitor neratinib was found to be cytoprotective in a glomerular cell culture model resembling the in‐vivo situation of glomerular damage [87]. Other drug repositioning studies identified AZD5438, a CDK2 kinase inhibitor, for the treatment of cisplatin‐induced acute kidney injury (AKI) [88] or compounds interfering with molecular mechanisms in the context of nephropathic cystinosis [89]. Interestingly, autosomal‐dominant polycystic kidney disease (ADPKD) is a prime target indication for drug repositioning approaches in the field of nephrology. This is probably due to the fact that the only currently approved drug tolvaptan, a selective vasopressin V2 receptor antagonist, cannot fully halt disease progression and is also associated with significant side effects. The high medical need to find and develop novel therapeutic strategies for ADPKD is imminent. Both experimental screening approaches [64, 90] and computational drug repositioning methods [91, 92] have been performed in recent years to identify compounds beneficially interfering with key molecular pathways in the development and progression of ADPKD. Key molecular mechanisms and potential drug targets of ADPKD have recently also been reviewed by Zhou and Torres [93]. This review on drug repurposing in ADPKD is in fact one of the first manuscripts that has been published in the special section on drug repurposing published by Kidney International [94]. Other publications in this series address drug repositioning candidates in the context of podocyte dysfunction [95], therapeutic options for proximal tubulopathies [96], as well as small molecules for the treatment of nephronophthisis and related renal ciliopathies [97]. Despite the fact that long lists of potential treatment options are presented for individual renal diseases, not all will make their way to the stage of being tested in clinical trials or all the way to the clinic.

We have recently identified clopidogrel as a promising therapeutic option for patients with FSGS following a computational network‐based drug repositioning approach and subsequent preclinical validation [98, 99]. Clopidogrel significantly reduced proteinuria levels in the adriamycin mouse model for FSGS and also ameliorated histopathological damage in renal tissue. Due to these positive in vivo data and clopidogrel's favorable safety profile, it appears as an attractive option for testing in human clinical trials. We have therefore set up the ClopiD4FSGS clinical trial to determine whether the effect that has been observed in the preclinical setting also holds true in the human setting [100]. Clopidogrel is indicated for a number of CVDs and specifically approved for atherosclerosis, myocardial infarction, peripheral arterial disease, and stroke.

Next to anti‐platelet drugs, other standard‐of‐care compound classes for patients with CVD include anti‐hypertensives, statins, beta‐adrenergic blockers, calcium channel blockers, or cardiac anti‐arrhythmic drugs. There are roughly a thousand different CVD entities within the MeSH ontology and for a large number of diseases there are still no effective treatments available. Lal et al. focused on atrial fibrillation, a CVD with poor treatment options, and used a transcriptomics‐based systems biology approach to computationally screen for drugs beneficially interfering with dysregulated molecular processes in atrial fibrillation. The anti‐diabetic medication metformin was among the top compound hits and was subsequently validated in the preclinical setting [101]. Wu et al. also made use of transcriptomics data to identify novel treatment options for patients with hyperlipidemia and hypertension. The strength of this study comes from the fact that they were able to assess the impact of top‐ranked compounds on LDL cholesterol levels, a marker for hyperlipidemia, and systolic blood pressure, a marker for hypertension, using EHR data from two large cohort studies [102]. Currently approved drugs for the treatment of hyperlipidemia and hypertension were found to significantly reduce the two parameters and served as positive controls for this approach. A number of new compounds beneficially impacting these two parameters were in addition identified. Although it was not a prospectively planned clinical trial, the approach nevertheless is a very efficient way to identify compounds with beneficial impacts on relevant parameters in the human setting of CVD. Another compound that was recently identified with a beneficial impact on blood pressure levels is 5‐aminosalicylic acid. This compound positively influences gut energy metabolism and microbiota dysbiosis, two processes that have previously been linked to hypertension on a mechanistic level [103]. Last but not least, there is the aforementioned case of colchicine that was initially developed for the treatment of gout and is currently approved in the US for CVD. A thorough review of drug repurposing options in the context of CVD was recently published by Abdelsayed et al. [104] with Ghosh et al. discussing drug repurposing opportunities in the context of stroke intervention with a focus on compounds in clinical testing [105].

But there is also the other way round, that is, the repositioning of drugs that are approved for CVDs for other indications outside the cardiovascular space. Tripathi et al., for example, discuss the role of statins as anti‐cancer therapeutics due to their effect on apoptotic processes via the BCL2 signaling cascade which subsequently has an impact on p53 signaling, mechanisms that are often dysregulated in the context of tumor development and progression [106]. Cancer is also one of the therapeutic areas that is discussed as an indication field for certain CVD compounds in the review by Ishida et al. [107]. Other indications in which CVD drugs are evaluated are, for example, cirrhosis, hemangioma, osteoporosis, Marfan's syndrome, or certain kidney diseases.

### 4.1 Repurposed Compounds in Running Clinical Phase III Trials for Kidney and Cardiovascular Disease

**FIGURE 3:**
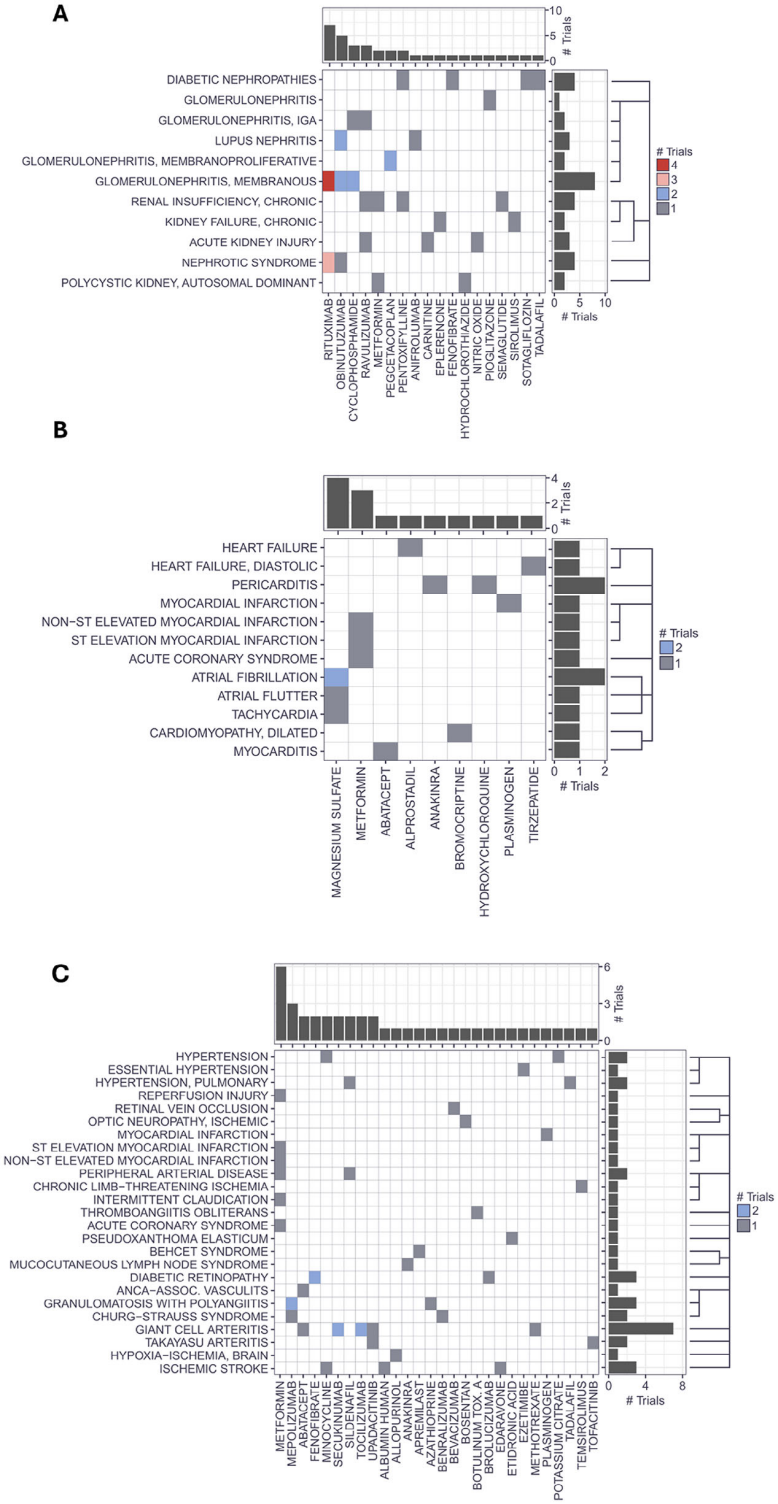
Running Phase III clinical trials in the cardiorenal space testing compounds approved for other indications. Running Phase III clinical trials for kidney diseases (A), heart diseases (B), and vascular diseases (C) testing compounds initially approved for different indications. Colors indicate the number of trials for a disease–drug pair. Bar plots at the top and to the right of the heatmaps show the sum of trials for a drug or disease, respectively. Drugs are ordered from left to right by the number of trials in decreasing order. Diseases are grouped based on MeSH hierarchy as schematically indicated by the dendrograms to the right.

We used Delta4's Hyper‐C software platform to extract information on running Phase III clinical trials in the context of renal and CVDs in June 2024. Curated phenotype and drug catalogs based on MeSH and ChEMBL were used to mine clinical trial information on ongoing Phase III clinical trials. Ongoing clinical trials included trials with one of the following statuses: “Active, not recruiting”, “Recruiting”, “Not yet recruiting”, or “Enrolling by invitation”. Only clinical trials of type “intervention” have been included, which are testing at least one drug. Renal and cardiovascular neoplasm disease entities have been removed from the analysis. Information regarding approved indications for drugs in the catalog has been extracted from FDA drug labels. Drug repositioning cases in the renal context were considered if the drug that is tested in a Phase III clinical trial for a dedicated renal disease has already been approved for at least one other indication outside the area of nephrology without any approval for any nephrological disease. The same procedure was done to list all drug repositioning cases in the cardiovascular space. We identified 18 unique drugs being tested for 11 renal diseases which are approved for diseases outside the field of nephrology (Figure 3A). CVDs were separated into vascular diseases and heart diseases. We identified 9 unique drugs being tested for 12 heart diseases which are approved for diseases outside the field of cardiovascular (Figure 3B). Similarly, we identified 28 unique drugs being tested for 25 vascular diseases, which are approved for diseases outside the field of CVDs (Figure 3C).

Almost 50 unique drugs in clinical Phase III testing for renal and CVDs are approved for indications outside the renal and cardiovascular area, respectively, highlighting that drug repositioning is a very promising approach to bring new therapeutic options to the market and thus to the patient in the end.

## 5 Conclusion and Future Perspective

Drug repositioning is a hot topic at the moment and the list of positive drug repositioning cases is getting longer. The systematic identification of drug repositioning opportunities, however, is still a young research field and it has become evident that bringing a repositioned product to the market involves much more than just the initial discovery. Whereas academic research groups are key stakeholders in developing new methods or generating new datasets that lead to a better understanding of disease pathobiology and drug mechanism of action, thus forming the basis for the identification of novel repositioning opportunities, they often lack the capabilities of further drug development. Big pharma companies, on the other hand, are primarily focusing on their own products whilst often missing out on opportunities outside their core business areas. In the end, it may be public–private partnerships, small biotech, and “techbio” companies who are the innovators in the drug repositioning space, coming up with novel opportunities and the capabilities for further development. After the positive completion of Phase II clinical trials, pharmaceutical companies are typically in‐licensing these assets to run the pivotal Phase III clinical trials and bring the rediscovered products to the market.

Despite some incentives from the regulatory perspective like the Orphan Drug Designation pathway or the 505(b)(2) pathway, some hurdles remain to make drug repositioning programs even more attractive. There is, for example, still a lack of funding for Phase II clinical trials for repurposed compounds as this is often considered not innovative enough. Starting a Phase II clinical trial is thus associated with a significant risk of failure. Having access to large population datasets for in‐silico validation of initial repurposed drug candidates would help to further reduce the risk as exemplified in the context of hyperlipidemia and hypertension by Wu et al. who had access to two large cohorts for retrospective data analysis and data validation [102]. Maturation of repurposed drug candidates, that is, re‐dosing or re‐formulation of existing drugs for the new indication is often necessary, both from the perspective of efficacy as well as from the IP and business perspective to bring a patent‐protected product to the market. The development of drug combinations is a very attractive way to bring highly innovative and effective products to the market and leverage a certain period of market exclusivity [108].

Overall, for people suffering from a rare disease, drug repositioning is the option with the highest chance for a treatment becoming available within a reasonable time. With the growing number of successful drug repositioning programs, we strongly believe that this is not merely a passing trend but a viable concept for the long‐term to bring new therapies to the market in a fast and efficient way.

## Author Contributions

P.P. conceptualized and planned this viewpoint article. P.P., M.L., K.K.I, and K.K. wrote the first draft of the manuscript. All authors contributed input, reviewed, and edited the manuscript. All authors approved the final draft.

## Conflicts of Interest

Paul Perco, Matthias Ley, Kinga Kęska‐Izworska, Dorota Wojenska, Enrico Bono, Samuel M. Walter, and Lucas Fillinger are employees of Delta 4 GmbH. Klaus Kratochwill is co‐founder of Delta4 GmbH.

## Data Availability

The authors have nothing to report.
